# Supplementary material for: Interaction of S100A6 Protein with the Four-Helical Cytokines
Source: Biomolecules. 2023 Sep 4;13(9):1345. doi: 10.3390/biom13091345 (PMC10526228; doi:10.3390/biom13091345)
Supplement: Supplementary file 1 [file biomolecules-13-01345-s001.zip › biomolecules-2554899-supplementary.pdf]

Supplementary Materials

Interaction of S100A6 Protein with the Four-Helical Cytokines

Alexey S. Kazakov [1], Evgenia I. Deryusheva [1], Victoria A. Rastrygina [1], Andrey S. Sokolov [1], Maria E. Permyakova [1], Ekaterina A. Litus [1], Vladimir N. Uversky [1,2,3,]*, Eugene A. Permyakov [1] and Sergei E. Permyakov [1,]*

[1] Pushchino Scientific Center for Biological Research of the Russian Academy of Sciences, Institute for Biological Instrumentation, Institutskaya str., 7, Pushchino, Moscow Region 142290, Russia; fenixfly@yandex.ru (A.S.K.); janed1986@ya.ru (E.I.D.); certusfides@gmail.com (V.A.R.); 212sok@gmail.com (A.S.S.); mperm1977@gmail.com (M.E.P.); ealitus@gmail.com (E.A.L.); epermyak@yandex.ru (E.A.P.)

[2] Department of Molecular, Morsani College of Medicine, University of South Florida, Tampa, FL 33612, USA

[3] USF Health Byrd Alzheimer's Research Institute, Morsani College of Medicine, University of South Florida, Tampa, FL 33612, USA

* Correspondence: vuversky@usf.edu (V.N.U.); permyakov.s@gmail.com (S.E.P.); Tel.: +7-(495)-143-7740 (S.E.P.); Fax: +7-(4967)-33-05-22 (S.E.P.)

Supplementary Tables

Table S1. The cytokine samples studied with regard to affinity to Ca^{2+}-loaded S100A6 in the present work: the panel of the cytokines from [47] is extended by Flt3L, SCF, and IL-19.

Full name	*Abbreviation*	*UniProt ID*	*Manufacturer*	*Cat. number*	*Source*
Short-chain cytokines					
Fms-related tyrosine kinase 3 ligand	Flt3L	P49771	PeproTech	300-19	*E.coli*
Granulocyte-macrophage colony-stimulating factor	GM-CSF	P04141	PeproTech	300-03	*E.coli*
Interleukin-2	IL-2	P60568	PeproTech	AF-200-02	*E.coli*
Interleukin-3	IL-3	P08700	SCI-Store (Russia)	PSG160-10	CHO
Interleukin-4	IL-4	P05112	PeproTech	AF-200-04	*E.coli*
Interleukin-5	IL-5	P05113	PeproTech	200-05	*E.coli*
Interleukin-7	IL-7	P13232	SCI-Store (Russia)	PSG240-10	CHO
Interleukin-9	IL-9	P15248	PeproTech	200-09	*E.coli*
Interleukin-13	IL-13	P35225	PeproTech	200-13	*E.coli*
Interleukin-15	IL-15	P40933	PeproTech	200-15	*E.coli*
Interleukin-21	IL-21	Q9HBE4	PeproTech	200-21	*E.coli*
Macrophage colony-stimulating factor 1	M-CSF	P09603	PeproTech	300-25	*E.coli*
Stem cell factor, soluble form	SCF	P21583	PeproTech	300-07	*E.coli*
Thrombopoietin	THPO	P40225	SCI-Store (Russia)	PSG090-10	CHO
Thymic stromal lymphopoietin	TSLP	Q969D9	PeproTech	300-62	*E.coli*
Long-chain cytokines					
Chorionic somatomammotropin hormone 1	PL	P0DML2	R&D Systems	5757-PL/CF	CHO
Granulocyte colony-stimulating factor	G-CSF	P09919	Pharmstandard (Russia)	n/a	*E.coli*
Growth hormone	GH	P01241	PeproTech	AF-100-40	*E.coli*
Growth hormone variant	GH-V	P01242	R&D Systems	7668-GH/CF	*E.coli*
Interleukin-12	IL-12	P29459* & P29460	PeproTech	200-12H	HEK293
Interleukin-23	IL-23	Q9NPF7* & P29460	PeproTech	200-23	Hi-5
Interleukin-27	IL-27	Q8NEV9* & Q14213	PeproTech	200-38	HEK293
Interleukin-31	IL-31	Q6EBC2	PeproTech	200-31	E.coli
Interleukin-35	IL-35	P29459* & Q14213	PeproTech	200-37	HEK293
Leptin	LEP	P41159	PeproTech	AF-300-27	E.coli
Prolactin	PRL	P01236	PeproTech	100-07	E.coli
Interferons/IL-10					
Interferon α-2	IFN-α2	P01563	Vector-Medica (Russia)	n/a	E.coli
Interferon γ	IFN-γ	P01579	Pharmaclon (Russia)	n/a	E.coli
Interferon ω-1	IFN-ω1	P05000	PeproTech	300-02J	E.coli
Interleukin-10	IL-10	P22301	PeproTech	AF-200-10	E.coli
Interleukin-19	IL-19	Q9UHD0	PeproTech	200-19	E.coli
Interleukin-20	IL-20	Q9NYY1	PeproTech	200-20	E.coli
Interleukin-22	IL-22	Q9GZX6	PeproTech	200-22	E.coli
Interleukin-24	IL-24	Q13007	PeproTech	200-35	CHO
Interleukin-26	IL-26	Q9NPH9	R&D Systems	1375-IL/CF	E.coli

* denotes the chain used for SCOP 2 [49] family assignment
n/a, not applicable

Table S2. The PDB [60] entries or AlphaFold2 [62] predictions used for structural modelling of the complexes of Ca^{2+}-loaded human S100A6 dimer (chains A, B of PDB entry 1K9K [61]) with the four-helical cytokines using ClusPro docking server [59].

Cytokine	*PDB entry*	*Chain*	*Method*	*Comments*
Short-chain cytokines				
EPO	1BUY	A	NMR	N24K, N38K, N83K substitutions
Flt3L	1ETE	A	X-ray	E41D substitution; homodimeric structure
GM-CSF	1CSG	A	X-ray	
IL-2	1IRL	A	NMR	F42A substitution
IL-3	6NMY	I	X-ray	W6Y substitution; complex with the receptor chains
IL-5	1HUL	A	X-ray	dimeric structure
IL-9	-	-	AlphaFold2	
IL-13	1GA3	A	NMR	
IL-15	4GS7	A	X-ray	complex with the receptor chains
IL-21	2OQP	A	NMR	
SCF	1EXZ	A	X-ray	homodimeric structure
THPO	1V7M	V	X-ray	complex with a neutralizing antibody fragment; only the structured fragment 22-184 was used for the docking
Long-chain cytokines				
CLCF1	-	-	AlphaFold2	
CNTF	-	-	AlphaFold2	
**CT-1**	-	-	AlphaFold2	
G-CSF	1GNC	A	NMR	
GH	-	-	AlphaFold2	
GH-V	-	-	AlphaFold2	
IL-11	6O4O	A	X-ray	
IL-31	-	-	AlphaFold2	
LEP	1AX8	A	X-ray	W100A substitution
PRL	1RW5	A	NMR	
Interferons/IL-10				
IFN-β	1AU1	A	X-ray	
IFN-ω1	3SE4	B	X-ray	N83Q substitution; complex with the receptor chains
IL-10	2ILK	A	X-ray	homodimeric structure
IL-19	1N1F	A	X-ray	F157S substitution
IL-20	4DOH	A	X-ray	complex with the receptor chains
IL-22	1M4R	A	X-ray	
IL-24	6DF3	C	X-ray	N34Q, N48Q, Y73H, N75Q substitutions; complex with the receptor chains
IL-26	-	-	AlphaFold2	

Table S3. The samples of the four-helical cytokines lacking specificity to S100A6, as evidenced by SPR experiments using cytokine as a ligand.

Full name	*Abbreviation*	*UniProt ID*	*Manufacturer*	*Cat. number*	*Source*
Short-chain cytokines					
Interleukin-4	IL-4	P05112	PeproTech	AF-200-04	*E.coli*
Interleukin-7	IL-7	P13232	SCI-Store (Russia)	PSG240-10	CHO
Macrophage colony-stimulating factor 1	M-CSF	P09603	PeproTech	300-25	*E.coli*
Thymic stromal lymphopoietin	TSLP	Q969D9	PeproTech	300-62	*E.coli*
Long-chain cytokines					
Chorionic somatomammotropin hormone 1	PL	P0DML2	R&D Systems	5757-PL/CF	CHO
Interleukin-6[#]	IL-6	P05231	PeproTech	200-06	*E.coli*
Interleukin-12	IL-12	P29459* & P29460	PeproTech	200-12H	HEK293
Interleukin-23	IL-23	Q9NPF7* & P29460	PeproTech	200-23	Hi-5
Leukemia inhibitory factor[#]	LIF	P15018	PeproTech	300-05	*E.coli*
Oncostatin-M[#]	OSM	P13725	PeproTech	300-10H	HEK293
Interferons/IL-10					
Interferon α-2	IFN-α2	P01563	Vector-Medica (Russia)	n/a	*E.coli*
Interferon γ	IFN-γ	P01579	Pharmaclon (Russia)	n/a	*E.coli*

[#] ref. [46]
* denotes the chain used for SCOP 2 [49] family assignment
n/a, not applicable

Table S4. Serum levels of S100A6 under normal and pathological conditions, according to the literature data.

Disorder	*S100A6 concentration*				*Reference*
	Disease		*Healthy control*		
	pg/ml	*pM*	*pg/ml*	*pM*	
Cholangiocarcinoma	2085.8 (2–13903.3) Pre-op 2197.7 (0–6817.8) Post-op	215.4 (0.2–1436.1) Pre-op 227.0 (0–704.2) Post-op	2468.4 (0–10841.5)	255.0 (0–1119.9)	[68]
Gastric cancer	52.96±13.57	5.47±1.40	19.83±7.46	2.045±0.77	[78]
Pre-eclampsia	600	62	59800	6177	[70]
Non-small cell lung cancer	12760±651.8	1318±67.3	8434±408.2	871±42.2	[71]
Acute coronary syndrome	4360±2450	450±253	3970±2570	410±265	[72]

Table S5. The contact residues for the S100A6-cytokine complexes predicted using ClusPro docking server [59] (numbering is according to the PDB entries). The receptor-binding residues of the cytokines are marked in bold; the following receptors were considered: EPO receptor (PDB ID: 1CN4), Flt3L receptor (PDB ID: 3QS7), G-CSF receptor (PDB ID: 2D9Q), GH receptor (PDB ID: 3HHR), GM-CSF receptor subunit α (PDB entry 4RS1), IFN α/β receptors 1 and 2 (PDB ID: 3SE4), IL-2 receptor subunits α, β and γ (PDB entry 2B5I), IL-3 receptor subunit α (PDB ID: 5UV8), IL-5 receptor subunit α (PDB ID: 3VA2), IL-10 receptor α chain (PDB ID: 1J7V), IL-13 receptor α-1 chain and IL-4 receptor α chain (PDB ID: 3BPO), IL-15 receptor subunits α, β and γ (PDB ID: 4GS7), IL-20 receptor subunits α and β (PDB ID: 4DOH), IL-21 receptor (PDB ID: 3TGX), IL-22 receptor subunit α-1 (PDB ID: 3DGC), IL-22 receptor subunit α-1 and IL-20 receptor subunit β in complex with IL-24 (PDB ID: 6DF3), PRL receptor (PDB ID: 3D48), SCF receptor Kit (PDB ID: 2E9W).

Short-chain cytokines		
**S100A6 dimer**		**EPO**
Chain A	Chain B	
T43, I44, K47, L48, E52, R55, D59, I83, Y84, E86, A87, L88, K89	A2,D6	P2,R4,**D8**,R10,R162
**S100A6 dimer**		**Flt3L**
Chain A	Chain B	
R55,I83,E86		L26,L27,Y30,T33,R55,W56,R59,T62,V63,P90,F96
**S100A6 dimer**		**GM-CSF**
Chain A	Chain B	T10,Q11,W13,E14,V16,N17,Q20,E21,R23,R24,N27,K72,**L115**,V116,**I117**,**F119**
R55,D59,I83,Y84,E86,A87,K89		
**S100A6 dimer**		**IL-2**
Chain A	Chain B	
A2,Q7,K18,K40		K9,**L19**,**Q22**,**M23**,H79,L80,**R81**,P82,**D84**,**L85**,**N88**,**E95**
**S100A6 dimer**		**IL-3**
Chain A	Chain B	
I44,E52,R55,D59,I83		Y13,V14,**S17**,N51,R55,L82,P83,C84,**P86**,L87,**A88**,T89,A91,L118,E119,**N120**
**S100A6 dimer**		**IL-5**
Chain A	Chain B	
E52	A2	V33,H34,K35,N36,L39,**K79**,C82,G83,E85,**R86**,**R87**,V89,F92,Y95,F99,W107
**S100A6 dimer**		**IL-9**
Chain A	Chain B	
L42,T43,I44,K47,L48,E52,R55, I83,Y84,E86,A87	A2,D6	P87,Y88,K116,K118,R120,R123,G124,K125, I126
**S100A6 dimer**		**IL-13**
Chain A	Chain B	
L42,T43,I44,K47,E52,R55,D59, L80,I83,Y84,E86,A87,L88,K89	D6,G10	P15,S16,**T17**,**R74**,**G78**,S90,N126
**S100A6 dimer**		**IL-15**
Chain A	Chain B	
Q7,I44,I83,Y84,L88	D6,K18	**L52**,**E64**,N110
**S100A6 dimer**		**IL-21**
Chain A	Chain B	

L42,T75,G78,Y84,A87,L88	A2,D6,Q7,T43,V74,T75,Y84,L88	M5,Q6,G7,Q8,D9,**R10**,H11,N46,W49,**P84**,**S85**,R90,H94,R95,H127,L128,S129,S130
**S100A6 dimer**		**SCF**
Chain A	Chain B	
Y84	D6	P31,G32,D34,V35,L95,S98,F99,E103,R105,R118,D131,C132,V133
**S100A6 dimer**		**THPO**
Chain A	Chain B	L3,R4,S7,H14,F40,S41,L42,G43,R130,R134,F135,M137,L138,G140
I83,Y84,A87,K89	A2	
Long-chain cytokines		
**S100A6 dimer**		**CLCF1**
Chain A	Chain B	
I44,R55,D59,T75,A79,I83,Y84		L1,N2,R3,T4,G5,P184,H192,L193,H196,G197,F198
**S100A6 dimer**		**CNTF**
Chain A	Chain B	
Q8,G10,L11,A14,D59,Y84	Q8,G10,L11,A14,D59,Y84	A2,F3,T4,R14,R177,F178,S181,H182,Q183,T184,I186,A188,R189,S191,H192,Y193
**S100A6 dimer**		**CT-1**
Chain A	Chain B	
T43,I44,E52,R55,D59,L80,I83,Y84,E86,K89	A2,D6	S16,G70,S72,L81,P82,V83,H84,E85,R88,L89,A145,A146,N147,R148,G149,P150,R151,A152,E153,P155,A157
**S100A6 dimer**		**G-CSF**
Chain A	Chain B	
I44,R55,I83,Y84	A2,T75	Q71,F84,Q91,W119,M127,P129,Q132,P133,Q135,G136
**S100A6 dimer**		**GH**
Chain A	Chain B	
I44,R55,D59,T75,Y84	Q7,Q71	**H18**,**R64**,**D171**,**E174**,**R178**,**C182**,G187,**C189**,**G190**,F191
**S100A6 dimer**		**GH-V**
Chain A	Chain B	
R55,D59,R62,Q71,I83,Y84	D59,R62	F1,T3,R8,R18,E119,T123,W126,R127,T135,S188,C189,G190
**S100A6 dimer**		**IL-11**
Chain A	Chain B	R17,M50,A52,A54,L58,Q59,D156,W157,R160,L163,L164,T167,R168
R55,D59,R62,Q71,T75	R62,N63,N69,Q71,E72,T75,L82,E86	
**S100A6 dimer**		**IL-31**
Chain A	Chain B	S1,H2,L4,P5,V6,R7,L9,S12,D53,A54,Q55,P56,P57,N58,F93,Q94,D95,Q138,A139,T141
A2,C3,Q7,K18,T43,I83,Y84	A2,C3,P4,Q7,L11,K18,K40,K47	
**S100A6 dimer**		**LEP**
Chain A	Chain B	
A2,Q7,L11,K18	A2,Q7,T43	L39,D40,F41,I42,P43,H46,I48,K53,V113,A116,S117,Y119,R128,D135,W138
**S100A6 dimer**		**PRL**
Chain A	Chain B	
T43,I44,K47,E52,R55,D59,A79,L82,I83,E86,K89	Q71,T75	L1,P2,I3,C4,G6,G7,A8,A9,R10,C11,Q12,V13,T14,R16,D17,R21,R125,E128,I194,H195,N196
Interferons/IL-10		

**S100A6 dimer**		**IFN-β**
Chain A	Chain B	
D59,R62,T75,L82,I83	R62,N63,K64,Q71,T75,A79	F8,L9,R11,S12,F15,Q16,Q18,K19,W22,R27,V148,R152,Y155,N158
**S100A6 dimer**		**IFN-ω1**
Chain A	Chain B	
Q7,T43,I44,E52,R55,D59,A79,I83,Y84,E86,A87,L88,K89		**R14,H21**,Q22,R24,R25,S27,**P28,F29**,L30,**R35**,**D37**,Q42,M147,K151,F154,N158
**S100A6 dimer**		**IL-10**
Chain A	Chain B	
	K47	K125,F128,I136,A139,M140,E142,F143,F146,I150,Y153,M154,K157
**S100A6 dimer**		**IL-19**
Chain A	Chain B	
L42,I44,K47,L48,E52,R55,L56,D59,A79,L80,L82,I83,E86,A87,K89	D6,Q71	L1,R3,K46,L48,K55,N56,R95,Q96,C97,Q98,Q102,H104,C105,R106,Q107,R114
**S100A6 dimer**		**IL-20**
Chain A	Chain B	
I44,L48,E52,R55,D59,R62,Q71,T75,A79,L82,I83	R62,Q71,T75	S8,R60,H61,R64,**R100**,L101,H103,**A104,H105**,**M106**,T107,C108,H109,C110,M115
**S100A6 dimer**		**IL-22**
Chain A	Chain B	
L42,I44,I83,E86,A87,K89		F47,H48,G49,M52,S53,R55,E102,R110,D135,**L136**,**M139**,**R142**,N143
**S100A6 dimer**		**IL-24**
Chain A	Chain B	
T43,I44,K47,E52,R55,D59,I83,Y84,E86,A87,K89	A2,D6,Q7	**E44**,Q104,N106,F109,S110,R112,D113,S114,H116,R117,R124
**S100A6 dimer**		**IL-26**
Chain A	Chain B	
I44,E52,D59,I83,Y84,E86,A87,K89	D6	K47,K49,E109,K111,R115,R118,Y121,R122

Table S6. The literature data on concentrations of the S100A6-specific four-helical cytokines in the physiological fluids under normal and pathological conditions.

Cytokine	*Condition*	*Fluid*	*Cytokine concentration*				*Reference*
			Disease		*Healthy control*		
			pg/ml	*pM*	*pg/ml*	*pM*	
			Short-chain cytokines				
EPO#	#		*IU/l*	*pM*	*IU/l*	*pM*	
	Healthy	Serum	–	–	7.8 (5.9–10.3)	2.8 (2.1–3.7)	[79]
	Essential thrombocythemia	Serum	5.2 (<1–25)	1.9 (<0.36–9.1)	4–21	1.4–7.6	[80]
	Diabetic retinopathy (DR)	Serum	7.00 (non-proliferative DR)<br>9.95 (proliferative DR)	2.5 (non-proliferative DR)<br>3.6 (proliferative DR)	6.90	2.5	[81]
	Breast cancer	Nipple aspirate fluid	72.5 (30.8–91.4)	26.3 (11.2–33.1)	11.7 (2.5–33.5)	4.2 (0.9– 12.1)	[82]
		Serum	11.8 (3.1–31.6)	4.3 (1.1– 11.5)	9.2 (2.5–27.5)	3.3 (0.9– 10.0)	
		Milk	–	–	25.0 (4.9–45.2)	9.1 (1.8–16.4)	
	Polycythemia vera	Serum	3.4	1.2	2.6–18.5	0.9–6.7	[83]
	Secondary erythrocytosis		23.7	8.6			
	Metastatic malignant melanoma	Serum	31.85 (19.80–280.00)	11.5 (7.2–101.5)	25.25 (21.10–40.00)	9.2 (7.6– 14.5)	[84]
Flt3L	Fanconi anemia	Serum	1331±2350 (0–10815)	56±99 (0–456)	14±39 (0–152)	0.59±1.64 (0–6.4)	[85]
	Aplastic anemia		460±187 (223–887)	19±8 (9–37)			
	Myelodysplastic syndromes	Serum	< 100–5735	< 4.2–242	–	–	[86]
	Acute myeloid leukemia<br><br>Day during treatment: 1, 8, 15, 22<br><br>(FLI: sustained increase of soluble Flt3L concentrations from day 1 to day 22 of induction;<br>FLD: an increase of Flt3L concentrations between days 1 and 15, followed by a decrease until day 22 of induction;<br>FLL: stable, low Flt3L concentrations (<1000 pg/ml) between day 1 and day 22 of induction)	Serum	FLI: 2 (0–234), 724 (0–7750), 3673 (65–14284), 5753 (1390–16088);<br>FLD: 6 (0–177), 1229 (4–7666), 6019 (1217–11640), 684 (14–9428);<br>FLL: 0 (0–34), 60 (0–419), 124 (0–800), 81 (13–213)	FLI: 0.08 (0–9.87), 30.53 (0–326.79), 154.25 (2.74–602.31), 242.58 (58.61–678.37);<br>FLD: 0.25 (0–7.46), 51.82 (0.17 – 323.25), 253.80 (51.32–490.82), 28.84 (0.59–397.55);<br>FLL: 0 (0-1.43), 2.53 (0–17.67), 5.23 (0–33.73), 3.42 (0.55–8.98)	–	–	[87]
	Sjögren's syndrome	Serum	94.6 (without lymphoma)<br>144.1 (with lymphoma)<br>cut off 175	4.0 (without lymphoma)<br>6.1 (with lymphoma)<br>cut off 7.4	64.4	2.7	[88]
GM-CSF	Axial spondyloarthritis	Serum	150	10.36	62	4.28	[89]

	Systemic sclerosis	Serum	120.9±125.5	8.29±8.67	20.1±12.3	1.39±0.85	[90]
	Chronic myelogenous leukemia	Serum	3.9–55	0.27–3.8	–	–	[91]
	Rheumatoid arthritis	Plasma	366±61<br>(severe)<br>376±44<br>(moderate)	25.27±4.22<br>(severe)<br>25.97±3.04<br>(moderate)	174±18	12.02±1.24	[92]
	Systemic lupus erythematosus	Plasma	256±41	17.67±2.83			
	Spondyloarthropathy	Plasma	190±32	13.02±2.21			
	Rheumatoid arthritis	Synovial fluid	1300	89.78	–	–	
	Relapsing–remitting multiple sclerosis	Cerebrospinal fluid	14.9±10.2<br>(relapse)<br>4.3±6.7<br>(remission)	1.03±0.70<br>(relapse)<br>0.30±0.46<br>(remission)	0±0	0±0	[93]
		Serum	27.8±18.4<br>(relapse)<br>19.4±13.8<br>(remission)	1.92±1.27<br>(relapse)<br>1.34±0.95<br>(remission)	17.9±13.3	1.24±0.91	
	Healthy	Serum	–	–	38.3 (26.3–63.8)	2.64 (1.82–4.41)	[94]
IL-2	Myasthenia gravis	Serum	0.179	0.012	0.011	0.007	[95]
	Rheumatoid arthritis	Serum	3.52–18.07<br>(moderate-high disease activity group)<br>1.67–5.29<br>(low disease activity group)<br>2.49–6.25<br>(remission group)	0.23–1.17<br>(moderate-high disease activity group)<br>0.11–0.34<br>(low disease activity group)<br>0.16–0.41<br>(remission group)	–	–	[96]
	Alopecia areata	Serum	22.2±1.19	1.44±0.08	21.1±2.68	1.37±0.17	[97]
	Asthma	Serum	42.7±21.1	2.77±1.37	30.3±2.4	1.97±0.16	[98]
		Sputum	35.3±13.2	2.29±0.86	35.3±8.4	2.29±0.54	
IL-3	Homozygous beta-thalassemia (children)	Plasma	6.9±17.1	0.46±1.13	0.2±0.5	0.013±0.033	[99]
	Healthy	Serum	–	–	<12	<0.8	[94]
	Healthy	Serum	–	–	5.8<br>(men)<br>8.0<br>(women)	0.38<br>(men)<br>0.53<br>(women)	[100]
	Chronic schizophrenia	Serum	27.0±19.7	1.79±1.31	14.6±10.4	0.97±0.69	[101]
	Cancer	Serum	12 (10–15.5)	0.80 (0.66–1.03)	14.5 (12–17)	0.96 (0.80–1.13)	[102]
IL-5	Healthy	Serum	–	–	<3.1	<0.2	[94]
	Hepatocellular carcinoma	Serum	10.97 (0.29–17042.32)	0.83 (0.02–1.30)	–	–	[103]
	Acute asthmatic attack	Serum	4.84±1.68	0.37±0.13	0.32±0.26	0.024±0.020	[104]
	Asthma	Serum	9.5<br>(atopic)<br>8.1<br>(non-atopic)	0.72<br>(atopic)<br>0.62<br>(non-atopic)	4.4	0.33	[105]
		Serum	0–46.75	0–3.56	0–5.20	0–0.40	[106]
	Chronic obstructive pulmonary disease	Serum	6.4±3.2	0.49±0.24	N/D	N/D	[107]
IL-9	Hodgkin's lymphoma	Serum	up to 3350	up to 237	–	–	[108]
	Healthy	Serum	–	–	113.6 (men)<br>573.3 (women)	8.1 (men)<br>40.6 (women)	[100]
	Rheumatoid arthritis	Serum	4.77±3.618	0.34±0.26	1.22±0.706	0.09±0.05	[109]

	Systemic lupus erythematosus	Serum	12.26±25.235	0.87±1.79			
	Colon cancer	Plasma	1.29	0.09	2.53	0.18	[110]
	Atopic asthma	Bronchoalveolar lavage fluid	up to 10000 (median 600)	up to 710 (median 43)	<300	<21	[111]
	Asthma	Serum	5020	355	3910	277	[112]
	Inflammatory bowel disease	Serum	40±5	2.83±0.35	<6	<0.4	[113]
	Diffuse large B-cell lymphoma	Serum	1.43±0.64	0.10±0.05	0.82±0.15	0.06±0.01	[114]
	Allergic rhinitis	Serum	12.6 (5.03–24.11) (exposed to allergen)	0.9 (0.3–1.7) (exposed to allergen)	–	–	[115]
			4.83 (1.97–14.01) (not exposed to allergen)	0.3 (0.1–1.0) (not exposed to allergen)			
	Systemic sclerosis	Serum	84.6±76.0	5.99±5.39	40.4±41.7	2.86±2.95	[116]
	Systemic lupus erythematosus		50.7±52.0	3.59±3.68			
	Dermatomyositis		50.6±55.8	3.58±3.95			
	Atopic dermatitis		41.8±38.8	2.96±2.75			
IL-13	Asthma	Serum	0–157.10	0–9.93	0–5.40	0–0.38	[106]
	Healthy	Serum	–	–	11.2 (men) 14.1 (women)	0.8 (men) 1.0 (women)	[100]
	Insulin resistance	Serum	37.69±17.82	2.38±1.13	15.88±6.71	1.13±0.47	[117]
	Chronic rhinosinusitis with nasal polyps	Serum	0.98±1.56	0.06±0.10	0.34±0.16	0.024±0.011	[118]
	Acute asthmatic attack	Serum	50.85±27.30	3.22±1.73	8.10±5.40	0.57±0.38	[104]
	Ocular Behcet's disease (BD)	Serum	38.7±59.6 (active ocular BD) 9.21±2.03 (ocular BD in remission) 10.3±1.65 (non-ocular BD in remission)	2.45±3.77 (active ocular BD) 0.58±0.13 (ocular BD in remission) 0.65±0.10 (non-ocular BD in remission)	7.83±1.85	0.56±0.13	[119]
	Hepatocellular carcinoma	Serum	38.425 (7.73–539.93)	2.43 (0.49–34.14)	–	–	[103]
	Psoriatic arthritis	Serum	2 (2–2)	0.13 (0.13–0.13)	2 (2–6.2)	0.14 (0.14–0.44)	[120]
	Rheumatoid arthritis		10.2 (2–28.3)	0.64 (0.13–1.79)			
	Osteoarthritis		2 (2–5.8)	0.13 (0.13–0.37)			
	Psoriatic arthritis	Synovial fluid	5.8 (2–14.8)	0.37 (0.13–0.94)	–	–	
	Rheumatoid arthritis		6.7 (2–75.5)	0.42 (0.13–4.77)			
	Osteoarthritis		2 (2–3)	0.13 (0.13–0.19)			
	Systemic sclerosis	Serum	0.84±0.65	0.05±0.04	0.35±0.18	0.025±0.012	[121]
IL-15	Polymyositis/dermatomyositis	Serum	47.6±170 (active)	2.7±13.3 (active)	28.5±28.89	2.5±2.3	[122]
			25.15±240 (inactive)	1.97±18.79 (inactive)			
	Healthy	Serum	–	–	<2.1	<0.16	[94]
	Essential hypertension	Serum	88.7±18.7	6.94±1.46	–	–	[123]

			(severe organ damage)<br>55.2±6.9<br>(mild organ damage)<br>51.0±8.3<br>(no organ damage)	(severe organ damage)<br>4.32±0.54<br>(mild organ damage)<br>3.99±0.65<br>(no organ damage)			
	Kawasaki disease	Serum	11.5 (5.8)<br>(acute stage)<br>1.3 (0.9)<br>(subacute stage)	0.91 (0.45)<br>(acute stage)<br>0.10 (0.07)<br>(subacute stage)	0.9 (1.0)	0.07 (0.08)	[124]
	Prostate cancer	Serum	208.07±48.50<br>(early stage prostate cancer)<br>50.51±14.34<br>(benign prostatic hyperplasia)	16.28±3.79<br>(early stage prostate cancer)<br>3.95±1.12<br>(benign prostatic hyperplasia)	–	–	[125]
	Multiple sclerosis (MS)<br><br>(PPMS: primary progressive MS;<br>RRMS: relapsing remitting MS;<br>SPMS: secondary progressive MS;<br>IDS: isolated demyelinating syndrome)	Serum	4.2±1.36<br><br>5.56±3.6<br>(PPMS)<br>4.13±0.61<br>(RRMS)<br>4.26±0.73<br>(SPMS)<br>4.13±0.45<br>(IDS)	0.33±0.10<br><br>0.44±0.28<br>(PPMS)<br>0.33±0.05<br>(RRMS)<br>0.34±0.06<br>(SPMS)<br>0.33±0.04<br>(IDS)	–	–	[126]
	Inflammatory neurological diseases		1.95±0.41	0.16±0.03			
	Non-inflammatory neurological diseases		2.96±1.13	0.23±0.09			
	Multiple sclerosis	Cerebrospinal fluid	3.27±0.91	0.25±0.07	–	–	
	Inflammatory neurological diseases		2±0.47	0.16±0.04			
	Non-inflammatory neurological diseases		2.43±0.49	0.18±0.04			
	Ulcerative colitis (moderate to severe)	Serum	0–490	0–38	–	–	[127]
	Hepatocellular carcinoma	Serum	77.4±78	6.06±6.11	–	–	
	Bacteremic melioidosis	Serum	49.4<br>(12.4–338.8)	3.87<br>(0.98–26.52)	12.8<br>(<8.2–122.2)	1.00<br>(<0.64–9.57)	[128]
	Non-bacteremic melioidosis	Serum	31.3<br>(11.6–2743)	2.45<br>(0.91–214.74)			
IL-21	Dengue virus infection	Serum	147.2±200.4<br>(up to >1000)	9.2±12.5<br>(up to >62)	14.5±59.4	0.91±3.71	[129]
	Plaque-type psoriasis	Serum	15.6 (15.63–335.5)	0.98 (0.98–20.98)	66.33 (7.8–1621.3)	4.15 (0.49–101.4)	[130]
	**Kawasaki disease**	Serum	499.5<br>(<62.5–1544)	31<br>(>3.9–96.6)	<62.5 (<62.5–850)<br>(febrile controls)	<3.9 (<3.9–53.2)<br>(febrile controls)	[131]
SCF	Gastrointestinal stromal tumors	Serum	645	34.8	950	51.3	[132]
	Asthma	Serum	1010±37	54.5±2.0	799±33	43.1±1.8	[133]
	Aspirin-exacerbated respiratory disease	Serum	516.1±202.7	27.9±10.9	416.4±119.6	22.5±6.5	[134]
	Autism spectrum disorders	Serum	3410±920	184.0±49.7	3450±1060	186.2±57.2	[135]

	Cancer	Serum	807.5 (674.5–955)	43.6 (36.4–51.5)	976 (921– 1060)	52.7 (49.7–57.2)	[102]
	Alzheimer's disease	Serum	661.1±40.0	35.7±2.2	997.7±33.7	53.8±1.8	[136]
THPO	Cirrhosis	Serum	69±12	1.82±0.32	49±9	1.3±0.24	[137]
	Myelosuppression after intensification chemotherapy of acute leukemia in complete remission	Serum	–	18.46±9.70	–	0.84±0.40	[138]
	Aplastic anemia		–	16.03±9.44			
	Acute lymphoblastic leukemia		–	10.36±5.57			
	Malignant lymphoma		–	2.79±2.27			
	Multiple myeloma		–	3.34±0.20			
	Chronic lymphocytic leukemia		–	1.71±3.91			
	Myeloproliferative disorders		–	1.99±1.47			
	Acute myelogenous leukemia		–	2.27±1.25			
	Hypoplastic leukemia		–	2.76±2.23			
	Myelodysplastic syndrome		–	0.42±0.60			
	Liver cirrhosis		–	1.50±0.92			
	Idiopathic thrombocytopenic purpura		–	2.08±1.41			
	Thrombocytopenia	Serum	>500	>14.1	<100	<2.8	[139]
	Ovarian cancer	Serum	114 (25–435)	3.2 (0.7–12.3)	–	–	[140]
	Benign ovarian disease		74 (34–189)	2.1 (1.0–5.3)			
	Consumptive thrombocytopenia	Serum	63 (48–98)	1.8 (1.4–2.8)	7–99	0.2–2.8	[141]
	Hypoproliferative thrombocytopenia		706 (358–1546)	19.9 (10.1–43.6)			
				Long-chain cytokines			
CLCF1	Nephrotic syndrome	Serum	477 (349– 585)	21.4 (15.7–26.3)	–	–	[142]
	Focal segmental glomerulosclerosis	Serum	1670	75	0–1200	0–54	[143]
CNTF	Rheumatoid arthritis	Serum	0.0–19.5	0.0–0.85	0.0–8.5	0.0–0.37	[144]
	Amyotrophic lateral sclerosis	Serum	0–2559	0–111.60	0–27.2	0–1.19	[145]
	Neural inflammation		0–54.0	0–2.35			
	Focal epilepsy	Serum	7.9±2.6	0.34±0.11	4.1±2.1	0.18±0.09	[146]
	Epilepsy and comorbid depression		8.3±5.1	0.36±0.22			
	Major depressive disorder		6.4±2.8	0.28±0.12			
	Age-related cataract and primary open-angle glaucoma	Aqueous humor	39.9±26.2	1.74±1.14	57.2±25.6	2.49±1.12	[147]
		Lacrimal fluid	25.7±14.9	1.12±0.65	39.9±18.0	1.74±0.78	
		Serum	5.45±4.72	0.24±0.21	5.96±4.92	0.26±0.21	
	Autism spectrum disorders with intellectual disability	Serum	1–42.3	0.04–1.84	0–3.42	0–0.15	[148]
	Intellectual disability	Serum	0.2–2.6	0.008–0.11			
	Obesity + diabetes	Serum	1199 (760–1634)	52.29 (33.14–71.26)	330 (191–495)	14.39 (8.33–21.59)	[149]
	Obesity	Serum	811 (510–1683)	35.37 (22.24–73.39)			
CT-1	Healthy	Serum	–	–	319.81±133.35	15.07±6.28	[150]

					(baseline)<br>293.50±137.70<br>(low salt diet)<br>360.40±162.83<br>(high salt diet)	(baseline)<br>13.83±6.49<br>(low salt diet)<br>16.98±7.67<br>(high salt diet)	
	Obstructive sleep apnea syndrome	Serum	19.47	0.92	8.23	0.39	[151]
	Congestive heart failure		–	74.3–182.8	–	6.9–48.3	[152]
	Healthy		–	–	–	572±75	
	Aortic stenosis		–	33–86.3	–	6.9–48.3	
	Congestive heart failure		–	544±21 (mild)<br>656±53<br>(severe)	–	200–500	
	Acute myocardial infarction		–	108.1±15.1	–	29.5±3.6	
	Unstable angina		–	42.2–527.4	–	6.9–54.1	
	Hypertension		–	53.52±4.29	–	18.51±2.29	
	Hypertrophic cardiomyopathy		–	136.28±72.01	–	17.92±12.03	[153]
G-CSF	Healthy	Serum	–	–	45.5 (34–53.6)	2.4 (1.8–2.80)	[94]
	Healthy	Serum	–	–	70.3 (men)<br>93.7 (women)	3.7 (men)<br>4.9 (women)	[100]
	Acute stage of infection	Serum	731.8±895.0<br>(30–3199)	38.6±47.1<br>(1.6–168.5)	25.3±19.7<br><100 in all cases	1.33±1.03<br><5.27 in all cases	[154]
	Breast cancer	Serum	92.81±594.54	4.88±31.31	0.00	0.00	[155]
	Acute bacterial infection	Serum	799±1501	42±79	–	–	[156]
	Viral infection		58±34	3.1±1.8			
	Atypical pneumonia		60±33	3.2±1.7			
	Chronic myelogenous leukemia	Serum	150–2830	7.9–149.1	–	–	[91]
	Urothelial carcinoma	Serum	147	7.74	<37	1.95	[157]
	Substance use disorders	Plasma	1414.3<br>(1095.3–1733.2)	74.4<br>(57.6–91.2)	1999.6 (1650.2–2349.0)	105.3 (86.9–123.7)	[158]
	Major depressive disorder		934.8<br>(388.7–1480.8)	49.2<br>(20.4–78.0)	2007.5<br>(1661.5–2353.5)	105.7<br>(87.5–124.0)	
	Various disorders	Serum	46 to >2000	2.4 to >105.3	<30 to 163	<1.6 to 8.6	[159]
	Septic shock after surgery	Serum	up to 20 000	up to 1050	<100	<5.27	[160]
GH	Gastric cancer	Serum	3140±3120<br>(200–10900)	142±141<br>(9–493)	690±1600<br>(100–9000)	31±72<br>(4.5–406.7)	[161]
	Colorectal cancer		3010±2910<br>(200–9900)	136±132<br>(9–447)			
	Healthy	Serum	–	–	400–10000<br>(adult males)<br>1000–14000<br>(adult females)<br>10000–50000<br>(children)	18–452<br>(adult males)<br>45–633<br>(adult females)<br>452–2260<br>(children)	[162]
	Acromegaly	Serum	>1000	>45	<1000	<45	[163]
	Healthy	Serum	–	–	2400±2800<br>(boys)<br>2500±3100<br>(girls)	108±127<br>(boys)<br>113±140<br>(girls)	[164]
GH-V	Down syndrome pregnancy	Maternal serum	Decreased in week 8 to 10.<br>In week 10 to 13, was not reduced.	–	531<br>(8th week)<br>935<br>(9th week)<br>962	23.79<br>(8th week)<br>41.89<br>(9th week)<br>43.10	[165]

					(10th week)<br>1103<br>(11th week)<br>1204<br>(12th week)<br>1169<br>(13th week)	(10th week)<br>49.42<br>(11th week)<br>53.94<br>(12th week)<br>52.37<br>(13th week)	
	Pre-eclampsia	Maternal serum	23 076<br>(3473–94 256)	1030<br>(156–4220)	12 157<br>(2617–34 016)	545<br>(117–1520)	[166]
IL-11	Non–small cell lung cancer	Serum	193.80<br>(123.50–323.60)	10.12<br>(6.45–16.90)	44.51<br>(23.24–77.05)	2.32<br>(1.21–4.02)	[167]
	Chronic heart failure	Serum	up to 120	up to 6.27	up to 70	up to 3.66	[168]
	Various arthritides	Serum	90 (71–130)	4.70 (3.71–6.79)	–	–	[169]
		Synovial fluid	165 (121–260)	8.62 (6.32–13.58)			
	Pancreatic adenocarcinoma	Serum	43.2	2.26	24.6	1.28	[170]
	Thyroid-associated ophthalmopathy	Serum	66.6±25.42	3.48±1.33	38.14±10.49	1.99±0.55	[171]
IL-27	Systemic sclerosis	Serum	302.8<br>(101.6–1034.4)<br>385<br>(109–826.3)	6.33<br>(2.12–21.62)<br>8.05<br>(2.28–17.27)	104.2<br>(51–184.2)	2.18<br>(1.06–3.85)	[172]
	Chronic HIV-1 infection	Serum	2008.0±274.8<br>(<50 copies/ml group)<br>1468.7±172.3<br>(51–10,000 copies group)<br>1237.9±127.3<br>(10,001–100,000 copies/ml group)<br>1590.1±223.7<br>(>100,000 copies/ml group)	42.0±5.7<br>(<50 copies/ml group)<br>30.7±3.6<br>(51–10,000 copies group)<br>25.9±2.7<br>(10,001–100,000 copies/ml group)<br>33.2±4.7<br>(>100,000 copies/ml group)	2990.7±682.1	62.5±14.3	[173]
	Childhood immune thrombocytopenia	Serum	770.6	16.1	373.8	7.8	[174]
	Ocular Behcet's disease (BD)	Serum	320±57.5<br>(active ocular BD)<br>316±47.3<br>(ocular BD in remission)<br>305±32.4<br>(non-ocular BD in remission)	6.69±1.20<br>(active ocular BD)<br>6.60±0.98<br>(ocular BD in remission)<br>6.38±0.67<br>(non-ocular BD in remission)	347±50.1	7.25±1.05	[119]
	Ischemic heart disease	Serum	38.00±14.38<br>(acute myocardial infarction)<br>35.77±18.93<br>(unstable angina)	0.80±0.30<br>(acute myocardial infarction)<br>0.75±0.40<br>(unstable angina)	24.91±14.96	0.52±0.31	[175]
	Diabetic retinopathy	Serum	240.900<br>(42.224–617.810)	5.0<br>(0.9–12.9)	2712.310<br>(1005.375–5786.877)	56.68<br>(21–120)	[176]
	Type 1 diabetes mellitus	Serum	22.06	0.46	14.5	0.30	[177]

	Type 1 diabetes mellitus + Hashimoto's disease		27.82	0.59			
	Healthy	Serum	–	–	most 100–1000 minority >2000 4,4% >6000	most 2.1–20.9 minority >41.8 4,4% >125.4	[178]
IL-31	Paediatric atopic dermatitis	Serum	1600 (1457.8±770.4) (flare) 1040 (958.7±419.5) (quiescence)	101 (92±49) (flare) 66 (61±26) (quiescence)	220 (197.3±91.9)	14 (12±6)	[179]
	Osteoporosis	Serum	43.12±6.97	2.72±0.44	29.58±6.09	1.86±0.38	[180]
	Allergic rhinitis	Serum	4107.70±16961.51	259±1070	2195.55±9016.57	139±569	[181]
	Early axial spondyloarthritis	Serum	12.6±15.4	0.79±0.98	1.8±4.0	0.11±0.25	[182]
	Chronic kidney disease-associated pruritus	Serum	679.9±1112.3	42.9±70.2	57.3±65.1	3.6±4.1	[183]
	Atopic dermatitis	Serum	up to 10 000	up to 631	up to 1200	up to 76	[184]
	Endometrial cancer	Serum	165.80±39.03 (94.43–240.65) (before operation) 128.91±29.48 (53.54–187.54) (after operation)	10.47±2.46 (5.97–15.20) (before operation) 8.14±1.86 (3.38–11.84) (after operation)	77.24±25.85 (32.50–142.82)	4.87±1.63 (2.05–9.02)	[185]
IL-35	Thyroid cancer	Serum	48.8±7.8	1.06±0.17	–	–	[186]
	Thyroid adenoma		62.3±9.6	1.36±0.21			
	Acute myeloid leukemia	Serum	51 (28–88)	1.11 (0.61–1.92)	22.2 (10.5–36.8)	0.48 (0.23–0.80)	[187]
	Kawasaki disease	Serum	15.07±1.37	0.33±0.03	28.86±4.92	0.63±0.11	[188]
	Idiopathic inflammatory myopathies	Serum	119.5 (32.1–1074.5)	2.61 (0.70–23.43)	36.2 (1.5–86.5)	0.79 (0.03–1.89)	[189]
	Asthma	Sputum	4890 (2970–22750)	107 (65–496)	6010 (4090–30470)	131 (89–664)	[190]
	Idiopathic membranous nephropathy	Serum	174.87 (remission) 151.87 (no remission)	3.81 (remission) 3.31 (no remission)	–	–	[191]
	Severe obstructive sleep apnea	Serum	1032.41±363.51	22.51±7.93	99.55±80.48	2.17±1.76	[192]
LEP	Asthma	Serum	13810±10560	862±659	6320±5200	394±324	[193]
	Multiple sclerosis	Serum	–	50–6250	–	–	[194]
	Multiple sclerosis (MS) (RRMS: relapsing remitting MS; SPMS: secondary progressive MS; PPMS: primary progressive MS)	Serum	11135±6453 (RRMS) 30258±14435 (SPMS) 30301±12709 (PPMS)	695±403 (RRMS) 1890±901 (SPMS) 1890±793 (PPMS)	–	–	[195]
	Non–small cell lung cancer	Serum	8500±1600 (all patients) 10700±2500 (without weight loss) 7200±2100 (weight loss)	530±100 (all patients) 668±156 (without weight loss) 449±131 (weight loss)	12800±2400	799±150	[196]
	Obesity	Serum	44920±26490	2800±1650	21810±17110	1360±1070	[197]

			(women) 15150±9500 (men)	(women) 945±593 (men)	(women) 7310±5080 (men)	(women) 456±317 (men)	
	Obesity	Serum	11390±8790 (boys) 12640±9330 (girls)	711±549 (boys) 789±582 (girls)	4800±5250 (boys) 4810±4430 (girls)	300±328 (boys) 300±276 (girls)	[198]
	Obstructive sleep apnea syndrome	Serum	2070	129	1290	80	[151]
PRL	Healthy	Serum	–	–	<20000 (men) <25000 (nonpregnant women) 80000–400000 (pregnant women)	<870 (men) <1090 (nonpregnant women) 3490–17470 (pregnant women)	[12]
	Phenylketonuria	Serum	12000 (3000–75000)	524 (131–3280)	1400–24000 (females) 1600–10700 (males)	61–1050 (females) 70–467 (males)	[199]
	Systemic lupus erythematosus	Serum	17400±15100	760±660	6300±3200	275±140	[200]
	Rheumatic autoimmune diseases		13100±10300	572±450			
	Psoriasis	Serum	11290±8050 (3800–39600)	493±352 (166–1730)	7900±3930 (2800–18600)	345±172 (122–812)	[201]
	Breast cancer	Serum	5000–77900	218–3400	<20000	<873	[202]
	Type 2 diabetes mellitus	Serum	36840±6290	1610±275	10660±2450	466±107	[203]
			Interferons/IL-10				
IFN-β	Dengue	Serum	0–1490	0–74	–	–	[204]
	Autoimmune thyroid diseases	Serum	<4.328 (low IFN-β level group) >4.328 (high IFN-β level group)	<0.22 (low IFN-β level group) >0.22 (high IFN-β level group)	–	–	[205]
	Dermatomyositis	Serum	0–20	0–1	<4.1	<0.2	[206]
IFN-ω1	Dermatomyositis	Serum	0–120	0–5.95	0–60	0–2.98	[206]
IL-10	Healthy	Serum	–	–	12.6 (8.5–16.7)	0.68 (0.46–0.90)	[94]
	Healthy	Serum	–	–	2.7	0.14	[100]
	Melanoma	Serum	15–480	0.80–25.74	<3.0	<0.16	[207]
			8.75	0.47	<3.0	<0.16	
	Gastric cancer		27.52	1.48	<12	<0.64	
			>21.0	>1.13	<3.0	<0.16	
	Pancreatic cancer		>10.0	>0.54	N/D	N/D	
			>9.8	>0.53	3.0	0.16	
	Colorectal cancer		97.36	5.22	24.53	1.32	
			16.09	0.86	5.1	0.27	
	Hepatic cancer		12	0.64	6.3	0.34	
	Hodgkin lymphoma		>10	>0.54	7.1	0.38	
	Non-Hodgkin lymphoma		>7.98	>0.43	<5.0	<0.28	
	Lung cancer		>38.16	>2.05	32.55	1.75	
			21.4	1.15	9.2	0.49	
	Multiple myeloma		2390±820	128±44	340±150	18.23±8.04	
	B-cell lymphoma		26.0	1.39	18.0	0.97	
	Chronic lymphocytic leukemia		74	3.97	<13.68	<0.73	

	Psoriasis	Serum	89.5±18.7	4.8±1	117.2±23.4	6.29±1.25	[208]
	Rheumatoid arthritis	Serum	3.33–15.20 (moderate-high disease activity group) 3.07–7.91 (low disease activity group) 2.95–8.41 (remission group)	0.18–0.82 (moderate-high disease activity group) 0.16–0.53 (low disease activity group) 0.16–0.45 (remission group)	–	–	[96]
	Asthma	Serum	4.1±3.8	0.22±0.20	2.3±2.5	0.12±0.13	[98]
		Sputum	4.4±3.3	0.24±0.18	3.9±5.9	0.21±0.32	
	Kawasaki disease	Serum	92.89 ± 15.59	4.98±0.84	12.93 ± 3.30	0.69±0.18	[188]
IL-19	Pernicious Anemia	Serum	163.68±75.96	9.2±4.3	55.68±36.75	3.1±2.1	[209]
	Iron deficient anemia		35.49±40.97	2.0±2.3			
	Acne vulgaris	Serum	20–175	1.1–9.8	10–25	0.6–1.4	[210]
	Psoriasis	Serum	87	4.9	11	0.6	[211]
	Chronic obstructive pulmonary disease	Serum	91.9±20.4	5.2±1.1	63.2±16.8	3.5±0.9	[212]
	Atopic dermatitis (children)	Serum	61.1±105.7	3.4±5.9	2.1±5.9	0.12±0.33	[213]
	COVID-19	Serum	22.3 (18–32) (asymptomatic) 34.5 (26–56) (mild/moderate) 61.8 (44–84) (severe)	1.25 (1.01–1.80) (asymptomatic) 1.94 (1.46–3.14) (mild/moderate) 3.47 (2.47 – 4.72) (severe)	8.5 (8.2–9.2)	0.48 (0.46–0.52)	[214]
		Saliva	533.2 (402–703) (asymptomatic) 711.9 (526–827) (mild/moderate) 862.3 (690–1054) (severe)	29.9 (22.6–39.5) (asymptomatic) 40.0 (29.5–46.4) (mild/moderate) 48.4 (38.7 – 59.2) (severe)	102.1 (96–121)	5.7 (5.4–6.8)	
IL-20	Rheumatoid arthritis	Serum	30.2 (19.1–58.5)	1.72 (1.09–3.34)	13.1 (11–15.1)	0.75 (0.63–0.86)	[215]
	Psoriasis	Serum	20.418±20.587	1.16±1.17	2.703±3.043	0.15±0.17	[216]
	Rheumatoid arthritis	Synovial fluid	432100 (25400–1653000)	24650 (1450–94310)	0 (0–26700)	0 (0–1520)	[217]
	Non-small cell lung cancer	Serum	40.35 (29.86–63.81)	2.30 (1.70–3.64)	37.73 (32.49–50.80)	2.15 (1.85–2.90)	[218]
	Rheumatoid arthritis	Plasma	282 (134–438)	16.09 (7.65–24.99)	–	–	[219]
	Osteoarthritis		124 (110–147)	7.07 (6.28–8.39)			
	Rheumatoid arthritis	Plasma	89 (45–643)	5.08 (2.57–36.69)	71 (40–80)	4.05 (2.28–4.56)	[220]
	Systemic sclerosis	Serum	56.89±11.6	3.25±0.66	79.11±18.2	4.51±1.04	[221]
	Primary Raynaud's phenomenon		71.42±11.8	4.07±0.67			
IL-22	Psoriasis	Serum	284.1±49.7	16.96±2.97	425.4±82.8	25.4±4.94	[208]
	Psoriasis	Serum	71.600±150.759	4.27±9.00	2.358±2.486	0.14±0.15	[216]

	Psoriasis	Serum	7.8 (7.8–103.9)	0.47 (0.47–6.2)	4.6	0.27	[130]
	Non-small cell lung cancer	Serum	10.66 (1.44–70.34)	0.64 (0.09–4.20)	4.69 (0.35–12.29)	0.28 (0.02–0.73)	[218]
	Systemic sclerosis	Serum	68.3±44.4	4.09±2.65	59.52±16.7	3.55±1.00	[221]
	Primary Raynaud's phenomenon		63.1±11.8	3.77±0.70			
	Epithelial ovarian cancer	Plasma	34.19±4.58	2.04±0.27	22.1±1.71	1.32±0.10	[222]
	Benign ovarian epithelial neoplasm		23.1±1.78	1.38±0.11			
	Hepatocellular carcinoma	Serum	299.675 (5.99–1963.32)	17.89 (0.36–117.2)	–	–	[103]
	Multiple myeloma	Serum	75.5±63.3	4.51±3.78	8.1±5.7	0.48±0.34	[223]
	Low-grade squamous intraepithelial lesion	Serum	168.2	10.0	36.91	2.20	[224]
	High-grade squamous intraepithelial lesion		61.48	3.67			
	Breast cancer	Serum	317.53±14.33	18.96±0.86	62.67±19.11	3.74±1.14	[225]
	Type 2 autoimmune hepatitis	Serum	55.26	3.3	0.1	0.006	[226]
IL-24	Rheumatoid arthritis	Synovial fluid	2.25 (0–8.21)	0.12 (0–0.45)	–	–	[219]
	Spondyloarthritis		3.81 (0.30–11.0)	0.21 (0.017–0.606)			
	Osteoarthritis		0 (0–0.72)	0 (0–0.040)			
	Rheumatoid arthritis	Plasma	2210 (310–7290)	122 (17–401)	80 (0–3630)	4.4 (0–200)	
	Spondyloarthritis		2020 (0–6830)	111 (0–376)			
	Breast cancer	Serum	160.65±55	8.84±3.03	27.4±8.5	1.51±0.47	[227]
	Gastric cancer + *H. pylori* infection		76.2±16.27	4.2±0.9			
	Gastric cancer		72.5±17.84	3.99±0.98			
	H. pylori infection		32.78±12.96	1.8±0.71			
	Systemic lupus erythematosus	Serum	1375.70 (812.92–2096.54)	75.76 (44.76–115.45)	334.70 (129.13–634.91)	18.43 (7.11–34.96)	[228]
IL-26	Systemic lupus erythematosus	Serum	4040±11660	230±663	740±2020	42±115	[229]
	Neurosyphilis	Serum	6870	391	1670	95	[230]
	Severe asthma	Serum	1100±390	63±22	550±250	31±14	[231]
	Behçet's disease	Serum	4800±1320 (active) 2770±1026 (inactive)	273±75 (active) 158±58 (inactive)	310±140	18±8	[232]
	Crohn's disease with bacterial DNA	Serum	101600±25400	5780±1440	18900±11200	1070±637	[233]
	Rheumatoid arthritis	Serum	2430±3800	138±216	30±40	1.71±2.27	[234]
		Synovial fluid	46630±21920	2650±1250	–	–	
	Other inflammatory arthritis	Serum	8820±16320	502±928	30±40	1.71±2.27	
		Synovial fluid	55190±29240	3140±1660	–	–	

IU/l are used instead of pg/ml for EPO

Table S7. The concentration ranges of the S100A6-specific cytokines in the physiological fluids under normal and pathological conditions, extracted from the literature data collected in Table S6.

SCOP 2 family	*Cytokine*	*UniProt ID*	*Concentration range, pM*		*Comment*
			Disease	*Healthy control*	
Short-chain cytokines	EPO	P01588	0.36 – 101.5 (Blood)	0.9 – 16.4 (Blood)	
	Flt3L	P49771	0 – 678 (Blood)	0 – 6.4 (Blood)	
	GM-CSF	P04141	0.27 – 25.97 (Blood)<br>0 – 1.03 (CSF)<br>89.78 (SF)	1.24 – 12.02 (Blood)<br>0 (CSF)	
	IL-2	P60568	0.012 – 2.77 (Blood)<br>2.29 (Sputum)	0.007 – 1.97 (Blood)<br>2.29 (Sputum)	
	IL-3	P08700	0.46 – 1.79 (Blood)	0.013 – 1.13 (Blood)	
	IL-5	P05113	0 – 3.56 (Blood)	0 – 0.4 (Blood)	
	IL-9	P15248	0.09 – 355 (Blood)<br><710 (Bronchoalveolar lavage fluid)	0.06 – 277 (Blood)<br><21 (Bronchoalveolar lavage fluid)	
	IL-13	P35225	0 – 34.14 (Blood)<br>0.13 – 4.77 (SF)	0 – 1.13 (Blood)	
	IL-15	P40933	0 – 214.74 (Blood)<br>0.16 – 0.25 (CSF)	0.07 – 9.57 (Blood)	
	IL-21	Q9HBE4	0.98 – 96.6 (Blood)	0.49 – 101.4 (Blood)	
	SCF	P21583	34.8 – 184.0 (Blood)	22.5 – 186.2 (Blood)	
	THPO	P40225	0.42 – 43.6 (Blood)	0.2 – 2.8 (Blood)	
Long-chain cytokines	CLCF1	Q9UBD9	15.7 – 75 (Blood)	0 – 54 (Blood)	
	CNTF	P26441	0 – 111.6 (Blood)	0 – 21.59 (Blood)	Homodimer
	CT-1	Q16619	0.92 – 656 (Blood)	0.39 – 572 (Blood)	
	G-CSF	P09919	1.6 – 168.5<br>up to 1050 in lethal case<br>(Blood)	0 – 124 (Blood)	
	GH	P01241	9 – 493 (Blood)	4.5 – 2260 (Blood)	
	GH-V	P01242	156 – 4220 (Maternal Serum)	117 – 1520 (Maternal Serum)	
	IL-11	P20809	2.26 – 16.90 (Blood)<br>6.32 – 13.58 (SF)	1.21 – 4.02 (Blood)	
	IL-27	Q8NEV9* & Q14213	0.46 – 42 (Blood)	0.30 – 120 (Blood)	Heterodimer
	IL-31	Q6EBC2	0.79 – 631 (Blood)	0.11 – 139 (Blood)	
	IL-35	P29459* & Q14213	0.33 – 23.43 (Blood)<br>65 – 496 (Sputum)	0.03 – 2.17 (Blood)<br>89 – 664 (Sputum)	Heterodimer
	LEP	P41159	50 – 6250 (Blood)	80 – 1360 (Blood)	
	PRL	P01236	131 – 3400 (Blood)	61 – 1090 (Blood)<br>3490-17470 (Maternal Serum)	
Interferons/IL-10	IFN-β	P01574	0 – 74 (Blood)	<0.2 (Blood)	
	IFN-ω1	P05000	0 – 5.95 (Blood)	0 – 2.98 (Blood)	
	IL-10	P22301	0.16 – 128 (Blood)	0.12 – 18.23 (Blood)	Homodimer
	IL-19	Q9UHD0	1.01 – 9.8 (Blood)<br>22.6 – 59.2 (Saliva)	0.12 – 3.5 (Blood)<br>5.4–6.8 (Saliva)	
	IL-20	Q9NYY1	1.09 – 36.69 (Blood)<br>1450 – 94310 (SF)	0.15 – 4.56 (Blood)<br>0 – 1520 (SF)	

	IL-22	Q9GZX6	0.09 – 117.2 (Blood)	0.006 – 25.4 (Blood)	
	IL-24	Q13007	0 – 401 (Blood)<br>0 – 0.606 (SF)	0 – 200 (Blood)	
	IL-26	Q9NPH9	63 – 5780 (Blood)<br>2650 – 3140 (SF)	1.71 – 1070 (Blood)	

* denotes the chain used for SCOP 2 [49] family assignment
Blood, serum or plasma
SF, synovial fluid
CSF, cerebrospinal fluid
